# Phosphoantigen Burst upon Plasmodium falciparum Schizont Rupture Can Distantly Activate Vγ9Vδ2 T Cells

**DOI:** 10.1128/IAI.00446-15

**Published:** 2015-09-10

**Authors:** Marianne Guenot, Séverine Loizon, Jennifer Howard, Giulia Costa, David A. Baker, Shaneel Y. Mohabeer, Marita Troye-Blomberg, Jean-François Moreau, Julie Déchanet-Merville, Odile Mercereau-Puijalon, Maria Mamani-Matsuda, Charlotte Behr

**Affiliations:** a Univ. Bordeaux, CIRID, UMR 5164, Bordeaux, France; b Centre National de la Recherche Scientifique, CIRID, UMR 5164, Bordeaux, France; c Faculty of Infectious and Tropical Diseases, London School of Hygiene & Tropical Medicine, London, United Kingdom; d Department of Molecular Biosciences, The Wenner-Gren Institute, Stockholm University, Stockholm, Sweden; e CHU de Bordeaux, Immunology and Immunogenetic Laboratory, Bordeaux, France; f Institut Pasteur, Immunologie Moléculaire des Parasites, Paris, France

## Abstract

Malaria induces potent activation and expansion of the Vγ9Vδ2 subpopulation of γδT cells, which inhibit the Plasmodium falciparum blood cycle through soluble cytotoxic mediators, abrogating merozoite invasion capacity. Intraerythrocytic stages efficiently trigger Vγ9Vδ2 T-cell activation and degranulation through poorly understood mechanisms. P. falciparum blood-stage extracts are known to contain phosphoantigens able to stimulate Vγ9Vδ2 T cells, but how these are presented by intact infected red blood cells (iRBCs) remains elusive. Here we show that, unlike activation by phosphoantigen-expressing cells, Vγ9Vδ2 T-cell activation by intact iRBCs is independent of butyrophilin expression by the iRBC, and contact with an intact iRBC is not required. Moreover, blood-stage culture supernatants proved to be as potent activators of Vγ9Vδ2 T cells as iRBCs. Bioactivity in the microenvironment is attributable to phosphoantigens, as it is dependent on the parasite DOXP pathway, on Vγ9Vδ2 TCR signaling, and on butyrophilin expression by Vγ9Vδ2 T cells. Kinetic studies showed that the phosphoantigens were released at the end of the intraerythrocytic cycle at the time of parasite egress. We document exquisite sensitivity of Vγ9Vδ2 T cells, which respond to a few thousand parasites. These data unravel a novel framework, whereby release of phosphoantigens into the extracellular milieu by sequestered parasites likely promotes activation of distant Vγ9Vδ2 T cells that in turn exert remote antiparasitic functions.

## INTRODUCTION

In humans and nonhuman primates, the main peripheral blood γδT-cell subset expresses the Vγ9 and Vδ2 T-cell receptor (TCR) chains. This Vγ9Vδ2 T-cell subset accounts for 1 to 10% of total blood T lymphocytes and is expanded in patients upon infection by pathogens such as Plasmodium falciparum (1–5) or Mycobacterium tuberculosis (6) and in patients with lymphoid malignancies (7). In malaria patients, this expansion may play a dual role, both promoting pathology (3, 5) and contributing to the control of parasite density. Indeed, Vγ9Vδ2 T cells efficiently limit *in vitro* P. falciparum expansion by granulysin-dependent cytotoxicity (1, 8–10). In malaria patients, high levels of granulysin-expressing Vγ9Vδ2 T cells correlate with their *ex vivo* parasite-specific degranulation capacity, and elevated granulysin concentration in plasma suggests significant discharge during acute P. falciparum malaria (1). As a step toward a better understanding of how Vγ9Vδ2 T cells target parasites, we recently showed that the antiparasitic activity of Vγ9Vδ2 T cells targets the extracellular merozoites (1). The intraerythrocytic developmental stages, which appear insensitive to the antiparasitic effect (1), seem to potently trigger Vγ9Vδ2 T-cell activation and degranulation (1, 11–14). However, how precisely and which intraerythrocytic developmental stages activate Vγ9Vδ2 T cells still is unclear.

Vγ9Vδ2 T cells are activated by so-called phosphoantigens, which are nonpeptidic intermediate metabolites of the isoprenoid production pathway (15; recently reviewed in reference 16). The natural phosphoantigen (E)-4-hydroxy-3-methyl-but-enyl-pyrophosphate (HMBPP) is produced by the DOXP pathway and is 1,000 times more potent for specifically activating Vγ9Vδ2 T cells than the isopentenyl-pyrophosphate (IPP) molecule, which is produced by both the DOXP pathway and the mevalonate pathway (17, 18). Apicomplexa and, notably, Plasmodium spp. do not possess the mevalonate pathway and use the DOXP pathway to produce isoprenoids (19). Although it has been shown that Vγ9Vδ2 T-cell activation by P. falciparum extracts is abrogated by apyrase treatment (12), the involvement of the parasitic DOXP pathway has never been formally proven, and the potency of the bioactivity of parasitic phosphoantigens on Vγ9Vδ2 T cells has never been assessed.

In the case of tumor cells, it is well established that cell-to-cell contact is required for Vγ9Vδ2 T-cell activation, and, like cytotoxic αβ T cells, their activation may be triggered by the formation of a cytotoxic synapse during contact with an activating tumor target cell (20). Recent reports demonstrated a mandatory role for a B7-related butyrophilin (CD277/BTN3A) for the phosphoantigen-dependent activation of Vγ9Vδ2 T cells by tumor targets or mycobacterium-infected cells (21–24). One of the proposed models suggests that Vγ9Vδ2 T cells recognize BTN3A modifications induced by binding the phosphoantigens produced inside the target cells (22). However, phosphoantigens also can be released into the supernatant of microorganisms or infected cell cultures. Furthermore, soluble phosphoantigens can be pulsed onto the surface of noninfected presenting cells (25), which stimulate Vγ9Vδ2 T cells in a contact-dependent manner. This suggests that Vγ9Vδ2 T cells can be activated by soluble phosphoantigens at a distance from the producing cell.

In the case of P. falciparum, numerous studies have reported stimulation of Vγ9Vδ2 T cells by schizont extracts/infected red blood cell (iRBC) lysates (3, 11, 12, 14, 26), culture supernatants (26–29), and/or intact iRBCs cocultivated with Vγ9Vδ2 T cells (1, 5, 14, 30), suggesting that Vγ9Vδ2 T-cell contact with iRBC is dispensable. However, at this point, how P. falciparum intracellular stages activate Vγ9Vδ2 T cells is unknown.

To address these issues and to gain novel insights on Vγ9Vδ2 T-cell activation by P. falciparum, here we explored the expression of BTN3A by iRBC, the timing of phosphoantigen release by iRBC, and the involvement of the parasite DOXP pathway in phosphoantigen production, and we quantified the P. falciparum bioactivity for Vγ9Vδ2 T cells.

## MATERIALS AND METHODS

### P. falciparum culture.

FCR3 parasites were cultured in O^+^ red blood cells (RBCs) (Etablissement Français du Sang-EFS-Aquitaine, France) in complete parasite medium (CPM; RPMI 1640 supplemented with 10% human serum, gentamicin, glutamine, and hypoxanthine) and were regularly tested for the absence of Mycoplasma contamination (1). Parasitemia was assessed by hydroethidine staining (31) or examination of Giemsa-stained smears. Parasite cultures were synchronized by sorbitol (32) and/or heparin treatment (33). When required, midstage schizonts (around 38 to 40 h postinvasion [hpi]) were purified by gel flotation on gelofusin to >80% parasitemia (31).

### Generation of Vγ9Vδ2 T-cell lines.

Short-term lines (γδT-cell lines) were generated from healthy donor peripheral blood mononuclear cells (PBMCs; EFS Aquitaine, France) as previously described (1). Briefly, PBMCs were stimulated with 400 nM bromohydrin pyrophosphate (BrHPP) (IPH1101; Innate Pharma) in complete medium (RPMI 1640 supplemented with 10% decomplemented fetal calf serum, glutamine, and antibiotics) in the presence of 300 UI/ml interleukin-2 (IL-2). After 20 days, Vγ9Vδ2 T-cell purity was evaluated by flow cytometry (FACS Fortessa; BD Biosciences). γδT-cell lines were used when the percentage of Vδ2^+^ CD3^+^ cells was >80%, unless indicated otherwise. For each experiment, two γδT-cell lines, freshly generated in parallel from two distinct donors, were used.

### BTN3A staining.

PBMCs, γδT-cell lines, purified P. falciparum cultures and uninfected red blood cells (uiRBCs) were stained with 15 μg/ml anti-butyrophillin 3 (BTN3A) antibody (clone 20.1; kind gift from E. Scotet, Nantes, France) for 45 min at room temperature. Cells were washed once and stained with fluorescein isothiocyanate (FITC)-coupled goat anti-mouse antibody (Beckman-Coulter) for 1 h at room temperature. After washing, PBMCs and γδT-cell lines also were stained with anti-CD3-phycoerythrin (PE) antibody (Becton-Dickinson), and iRBCs were stained with hydroethidine (5 μg/ml) to discriminate iRBCs from uiRBCs. Surface marker expression was assessed by flow cytometry.

### CD107a degranulation assay.

The CD107a degranulation assay was performed as described previously (1). Briefly, 10^5^ cells of γδT-cell lines starved overnight of interleukin-2 (IL-2) or freshly collected PBMCs were incubated in the presence of stimulant and anti-CD107a-PE antibody (BD Bioscience) for 4 h. Cells were collected, washed, labeled with anti-Vδ2-FITC antibody (Beckman Coulter), and analyzed by flow cytometry. For transwell experiments, cell culture inserts (0.4-μm polycarbonate membrane; the Transwell system; Nunc, Roskilde, Denmark) were used by following the manufacturer's recommendations, with γδT cells seeded in the bottom of the wells in the presence of anti-CD107a-PE antibody and midstage schizonts (38 to 40 hpi) in the upper chamber. For antibody blocking experiments, γδT cells were preincubated for 1 h with anti-Vδ2 (clone immu389; Beckman Coulter), anti-BTN3A (clone 103.2; kind gift from D. Olive [21]), or anti-NKG2D (clone 149810; R&D Systems) antibody before stimulation as described previously (1).

### Preparation of iRBC supernatants.

Synchronized midstage schizont cultures (38 to 40 hpi) were washed and adjusted to 5% hematocrit in CPM. Culture supernatants (G, J, K, L, M, and S) were prepared from independent parasite cultures and collected 4 h later from a 4% parasitemia culture. Each culture supernatant was tested on at least two different γδT-cell lines, and at least two different supernatants were used in each assay. Rupture supernatants were collected from cultures at 1.5% parasitemia, when rupture was complete and reinvasion had occurred. Parasite stages were estimated from microscopic examination of Giemsa-stained smears. All of the collected supernatants were spun down at 870 × *g* for 5 min, filtered through a 0.22-μm Millipore filter, and frozen until use. As a control, supernatant from uiRBCs cultivated in parallel was collected using the same protocol. When indicated, supernatants were ultracentrifuged in a Beckman Optima L-100XP centrifuge in a 90Ti rotor at 197,000 × *g* for 3 h at 4°C. For pyrophosphatase treatment, we used 0.2 U/ml potato apyrase (Apy) (Sigma-Aldrich) for 1 h as described previously (34), followed by filtration using a 3-kDa-cutoff Centricon filter (Amicon) according to the manufacturer's instructions. Supernatant activity was tested by CD107a assay.

### Fosmidomycin treatment.

Young trophozoite iRBC cultures (20 hpi) were adjusted to 4% parasitemia, 5% hematocrit in CPM and incubated for 21 h with serial dilutions of fosmidomycin with or without the addition of 1 μM farnesyl pyrophosphate (both from Sigma-Aldrich). Duplicate supernatants of treated parasites were frozen until use. γδT-cell lines were treated similarly. In order to assess the effect of fosmidomycin on iRBC viability after 21 h of incubation, iRBC cultures were diluted to 1% parasitemia and 1% hematocrit and allowed to reinvade in fresh CPM. Cycle progression was assessed by Giemsa staining, and parasitemia was determined by hydroethidine staining as described above.

### Kinetics of phosphoantigen and PfHRP2 release.

Three independent cultures (A, B, and C) of synchronized iRBCs (14 hpi) were washed and adjusted to 1% parasitemia and 5% hematocrit in CPM and dispensed in 24-well plates. Cultures were unfed during the time of study. Supernatants from duplicate wells were collected at different time points, filtered through a 0.22-μm filter, and frozen until use. Supernatants from uiRBCs served as controls. Thawed supernatants were tested in duplicate for phosphoantigen bioactivity on two independent γδT-cell lines. iRBC rupture was assessed by monitoring parasite developmental stages using Giemsa-stained blood smears. P. falciparum histidine-rich protein 2 (PfHRP2) was measured in supernatants using a commercial enzyme-linked immunosorbent assay (ELISA) by following the manufacturer's instructions (Malaria Ag Celisa; Cellabs, Sydney, Australia).

### Treatment with the apicomplexan cGMP-dependent protein kinase inhibitor compound C2.

Synchronized schizont cultures (around 40 to 44 hpi) were washed once with RPMI, adjusted to 1% parasitemia and 5% hematocrit, and resuspended in CPM containing 2 μM compound 2, which was 4-[7-[(dimethylamino)methyl]-2-(4-fluorphenyl)imidazo[1,2-*a*] pyridin-3-yl]pyrimidin-2-amine, or its vehicle, dimethyl sulfoxide (DMSO) (35). iRBC supernatants were collected at various time points.

### Statistical analysis.

Statistical tests used are indicated in the figure legends when applied. When shown, error bars correspond to means ± standard deviations (SD).

## RESULTS

### Plasmodium falciparum-infected red blood cells do not express butyrophilin 3 and activate Vγ9Vδ2 T cells in the absence of contact.

**FIG 1:**
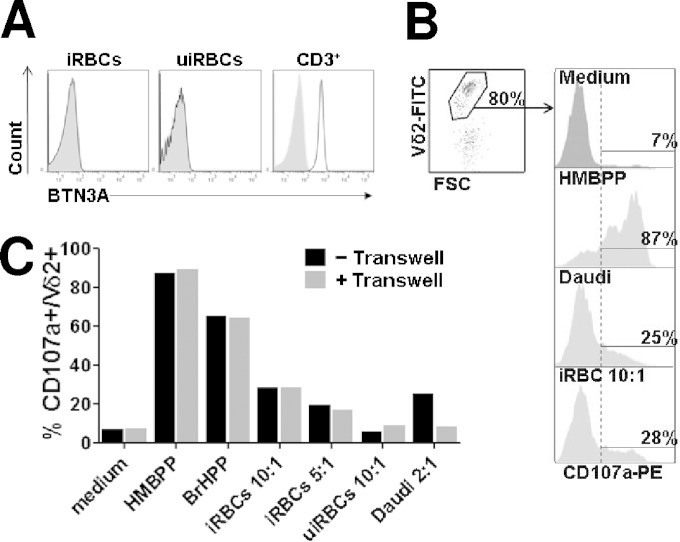
Plasmodium falciparum-infected RBC do not express butyrophilin 3 and activate Vγ9Vδ2 T cells without contact. (A) Plasmodium falciparum-infected (iRBCs) or uninfected red blood cells (uiRBCs) were incubated with anti-butyrophilin3 (BTN3A) antibody (black line) or with an isotypic control (light gray) and analyzed by flow cytometry. BTN3A expression in CD3^+^ PBMC also was analyzed. Shown are data of BTN3 labeling from one representative experiment out of three. (B) Gating strategy for CD107a degranulation test. Vγ9Vδ2 short-term lines (γδT-cell lines) were incubated with stimulants (medium, HMBPP, Daudi cells, or iRBC at a 10:1 target-to-effector ratio) and PE-labeled anti-CD107a antibody for 4 h, washed, and subsequently incubated with FITC-labeled anti-Vδ2 antibody. Degranulated cells are identified by flow cytometry as CD107a-positive cells within the Vδ2^+^ population. (C) Stimulants (100 nM HMBPP, 200 nM BrHPP) or target cells (iRBCs, uiRBCs, or Daudi cells at the indicated target/effector ratios) were either incubated for 4 h with Vγ9Vδ2 T cells or cultured in the upper chamber of the 0.4-μm polycarbonate transwell device. Vγ9Vδ2 T-cell degranulation was further assessed by CD107a assay after 4 h of incubation in contact with (gray bars) or physically separated from (black bars) stimulants as indicated. Shown are the results from one representative γδT-cell line (γδT-cell line 168) out of 4 (complete data are in Fig. S1 in the supplemental material). Midstage schizonts (38 to 40 hpi) were used as iRBCs.

We first asked whether P. falciparum iRBCs could activate Vγ9Vδ2 T cells by a mechanism similar to that of cancer cells, through BTN3A binding of intracellular phosphoantigens (21–24). Using flow cytometry we found that, contrary to T cells, neither uninfected RBC (uiRBC) nor iRBC expressed BTN3A (Fig. 1A). This suggests that iRBCs activate Vγ9Vδ2 T cells through an alternative mechanism, involving either another phosphoantigen presentation molecule on the iRBC surface or through recognition of phosphoantigens released by iRBCs into the microenvironment.

To examine whether contact of iRBCs with Vγ9Vδ2 T cells was mandatory for activation, Vγ9Vδ2 short-term lines (γδT-cell lines) were coincubated with iRBCs with or without physical separation by a transwell insert, and their activation was monitored using a CD107a degranulation assay (1). The gating strategy is illustrated in Fig. 1B, showing, as expected, that 87%, 25%, and 28% of Vγ9Vδ2 T cells activated with HMBPP, Daudi cells, and iRBC, respectively, expressed the CD107a marker of degranulation. Separation by a 0.4-μm transwell membrane abrogated Vγ9Vδ2 T-cell activation by the Daudi cell line, which is known to require cell-cell contact, and did not alter the response to soluble HMBPP. Interestingly, physical separation by the transwell did not abrogate or alter Vγ9Vδ2 T-cell reactivity to iRBCs (Fig. 1C; also see Fig. S1A in the supplemental material). This indicates that soluble mediators, released by mature iRBCs and diffusing freely across the 0.4-μm transwell membrane, activate Vγ9Vδ2 T cells in the absence of contact with iRBCs. This was confirmed by time-lapse confocal microscopy (see Fig. S1B), where almost all Vγ9Vδ2 T cells formed long-lived conjugates with Daudi cells, while iRBC-Vγ9Vδ2 conjugates were scarcely observed. Consistent with this, we did not detect conjugates between Vγ9Vδ2 T cells and iRBCs by flow cytometry. Altogether, these data suggest that triggering of Vγ9Vδ2 T-cell activation by iRBCs relies on soluble mediators released in the microenvironment.

### Molecules released in iRBC supernatant activate Vγ9Vδ2 T cells in a TCR- and BTN3A-dependent manner and have characteristics of phosphoantigens.

**FIG 2:**
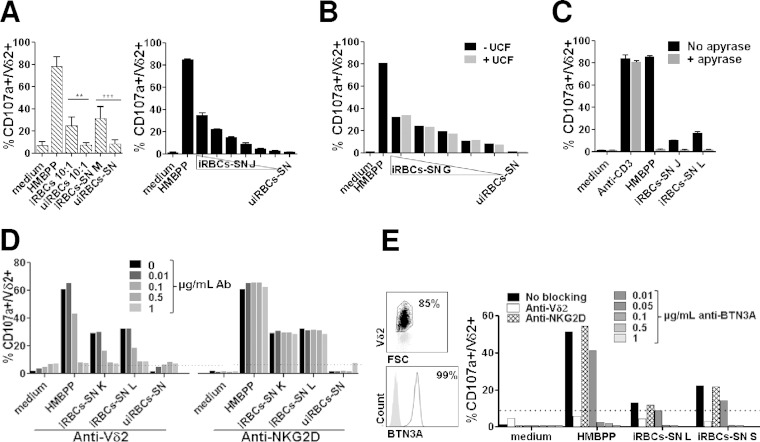
Vγ9Vδ2 T-cell activation is mediated by soluble phosphoantigen(s) in a TCR-dependent manner. The reactivity of Vγ9Vδ2 T cells toward iRBC culture supernatants (iRBC-SNs) collected from different parasite cultures (M, J, K, L, and S) at 4% parasitemia was assayed using the CD107a degranulation test. (A) Reactivity of Vγ9Vδ2 T cells from fresh PBMCs from 5 malaria-naive donors to intact iRBCs or uiRBCs at the indicated ratios was compared to the reactivity to the corresponding culture supernatants (left) (statistical significance is calculated using one-way analysis of variance [ANOVA]; **, *P* < 0.01; ***, *P* < 0.0001). Serial 2-fold dilutions of iRBC-SN J were assayed on Vγ9Vδ2 short-term lines (γδT-cell lines). (Right) Means ± SD from duplicates obtained from a representative experiment using one (γδT-cell line 226) out of two independently generated γδT-cell lines. (B) Reactivity of γδT-cell lines to iRBC-SN G was tested before (−UCF) and after (+UCF) ultracentrifugation at 197,000 × *g* on two γδT-cell lines (depicted are representative data obtained with γδT-cell line 151). (C) Vγ9Vδ2 T-cell degranulation was measured after incubation with medium alone, HMBPP (100 nM), and iRBC-SNs J and L and were left untreated (black bars) or treated (gray bars) with 0.2 U/ml apyrase at 37°C for 1 h, followed by 3-kDa-cutoff ultrafiltration (Centricon filter). Undiluted iRBC-SNs J and L were tested on γδT-cell lines. Anti-CD3 antibody was used as a phosphatase-insensitive activation control. Data shown are means from duplicates ± SD obtained from a representative experiment using one (γδT-cell line 244) out of two independently generated lines. (D) Vγ9Vδ2 T cells were preincubated with blocking antibodies against Vδ2 or NKG2D at concentrations indicated in the caption before activation with either HMBPP (5 nM) or iRBC-SNs K and L. As a negative control, supernatants from uninfected RBC (uiRBC-SNs) were collected and tested in parallel on two independent γδT-cell lines (shown is representative γδT-cell line 227). (E) Expression of BTN3A was assessed on γδT-cell lines after 20 days of expansion (left). Vγ9Vδ2 T cells then were preincubated with either 1 μg/ml anti-Vδ2 antibody or 1 μg/ml anti-NKG2D antibody or anti-BTN3A blocking antibody at the concentrations indicated before stimulation with either HMBPP (5 nM) or iRBC-SNs L and S (right). Shown are results obtained using representative γδT-cell line 387 (out of 3).

P. falciparum is known to produce HMBPP through the DOXP pathway, and iRBC extract has been shown to contain HMBPP; however, little is known about the release of phosphoantigens in iRBC culture supernatants. To gain insight on this aspect, we generated culture supernatants from iRBCs and examined their ability to activate Vγ9Vδ2 T cells. Consistent with transwell experiments, the iRBC supernatant induced Vγ9Vδ2 T-cell activation from fresh PBMCs in the same range as intact iRBCs (Fig. 2A, left). This shows that parasite supernatant activation of Vγ9Vδ2 T cells did not require their prior *in vitro* priming, expansion, or selection. Supernatants also induced degranulation of γδT-cell lines in a dose-dependent manner (Fig. 2A, right). Moreover, ultracentrifugation of iRBC supernatant did not alter its capacity to activate Vγ9Vδ2 T cells (Fig. 2B), indicating that the activating mediators are not carried by exosome-like vesicles released by iRBCs (recently described by Regev-Rudzki et al. [36]) that can cross the 0.4-μm transwell membrane.

Thus, we investigated whether the activating molecules released in the iRBC supernatant had the reported chemical characteristics of phosphoantigens (12). Supernatants ultrafiltered using 3-kDa-cutoff filters efficiently activated Vγ9Vδ2 T cells but lost this activity after a prior treatment with apyrase, similar to the prototypic phosphoantigen HMBPP (Fig. 2C). Apyrase treatment did not affect Vγ9Vδ2 T cells, as activation induced by an anti-CD3 antibody was insensitive to the addition of apyrase-treated supernatants. Therefore, the activating molecules released in the iRBC supernatant have a molecular mass lower than 3 kDa and are terminally pyrophosphorylated. Furthermore, activation of Vγ9Vδ2 T cells by iRBC supernatant was prevented using anti-Vδ2 blocking antibody in a dose-dependent manner, as was the activation by HMBPP (Fig. 2D). This indicates that iRBC supernatant, like HMBPP, stimulates Vγ9Vδ2 T cells in a TCR-dependent manner. NKG2D, an activating receptor expressed by Vγ9Vδ2 T cells, also could be involved in these mechanisms. However, including an anti-NKG2D blocking antibody in the activation test had no effect on Vγ9Vδ2 T-cell activation. γδT-cell lines expressed BTN3A after expansion (Fig. 2E, left). Activation of Vγ9Vδ2 T cells by phosphoantigens has been shown to be dependent on BTN3A expression by Vγ9Vδ2 T cells themselves (21). In line with this, Vγ9Vδ2 T-cell activation by iRBC supernatant was abrogated using neutralizing anti-BTN3A antibody (clone 103.2) (Fig. 2E, right). Thus, the activating molecule(s) released in the culture supernatant have the properties of phosphoantigens.

### The activating molecules released by iRBC are intermediates of the DOXP pathway.

**FIG 3:**
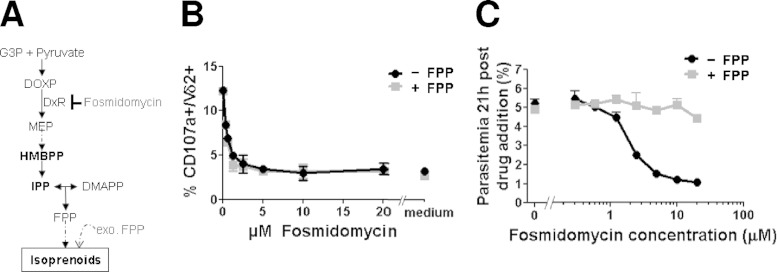
DOXP pathway inhibition abrogates activation of Vγ9Vδ2 T cells by iRBC-SN. (A) Simplified representation of the steps in the DOXP pathway relevant to our experimental protocol. G3P, glutaraldehyde 3 phosphate; DOXP, 1-deoxy-d-xylulose 5-phosphate; MEP, 2-*C*-methyl-d-erythritol 4-phosphate; HMBPP, (E)-4-hydroxy-3-methyl-but-2-enyl pyrophosphate; IPP, isopentenyl pyrophosphate; DMAPP, dimethylallyl pyrophosphate; FPP, farnesyl pyrophosphate; exo. FPP, exogenous FPP. Note that between MEP and HMBPP, several steps have been skipped. Endogenous products are in boldface characters. (B) Vγ9Vδ2 T-cell reactivity was tested toward iRBC-SNs collected from iRBC cultures treated for 21 h with fosmidomycin in the presence or absence of 1 μM FPP. Data shown are means ± SD of duplicates obtained from a representative experiment using γδT-cell line 266. (C) Effect of fosmidomycin treatment and exogenous FPP (1 μM) addition on parasite culture progression. IRBC cultures were treated during 21 h with fosmidomycin, with or without 1 μM FPP. Parasitemia after reinvasion was evaluated in triplicate wells. Controls in CPM alone without fosmidomycin are shown on the left.

In order to assess the contribution of the parasites' DOXP pathway to the production of iRBC-released bioactive molecules, we incubated iRBCs in the presence of fosmidomycin, which inhibits DOXP reductase, the first enzyme of this pathway (37) (Fig. 3A). Vγ9Vδ2 T-cell activation by iRBC culture supernatants was efficiently reduced by fosmidomycin in a dose-dependent manner (Fig. 3B, black line). However, as high doses of fosmidomycin specifically inhibit parasite cycle progression (Fig. 3C), the decrease of Vγ9Vδ2 T-cell activation might merely reflect a quantitative decrease of parasite maturation and a correlative diminution of phosphoantigen production. In order to circumvent this putative bias, fosmidomycin-treated parasite cultures were complemented with exogenous farnesyl pyrophosphate (FPP), which is produced downstream from the prototypical phosphoantigens HMBPP and IPP (Fig. 3A). In order to determine the optimum dose of FPP able to rescue parasitemia, we monitored the iRBC-treated cultures up to 42 h posttreatment and selected a dose of 1 μM FPP (see Fig. S2A in the supplemental material). While 1 μM FPP effectively restored the parasite cell cycle progression in the presence of up to 5 μM fosmidomycin for 21 h (Fig. 3C, gray line), it did not restore the Vγ9Vδ2 T-cell activation capacity of the fosmidomycin-treated culture supernatants (Fig. 3B, gray line). Of note, neither fosmidomycin nor FPP at the concentrations used affected Vγ9Vδ2 T-cell activation by HMBPP (see Fig. S2B). These results clearly demonstrate the obligatory link between phosphoantigen production by the parasite and Vγ9Vδ2 activation and indicate that the Vγ9Vδ2 activators are produced upstream from FPP and downstream from DOXP in the DOXP pathway.

### Vγ9Vδ2-stimulating molecules are released in the culture supernatant upon schizont rupture.

**FIG 4:**
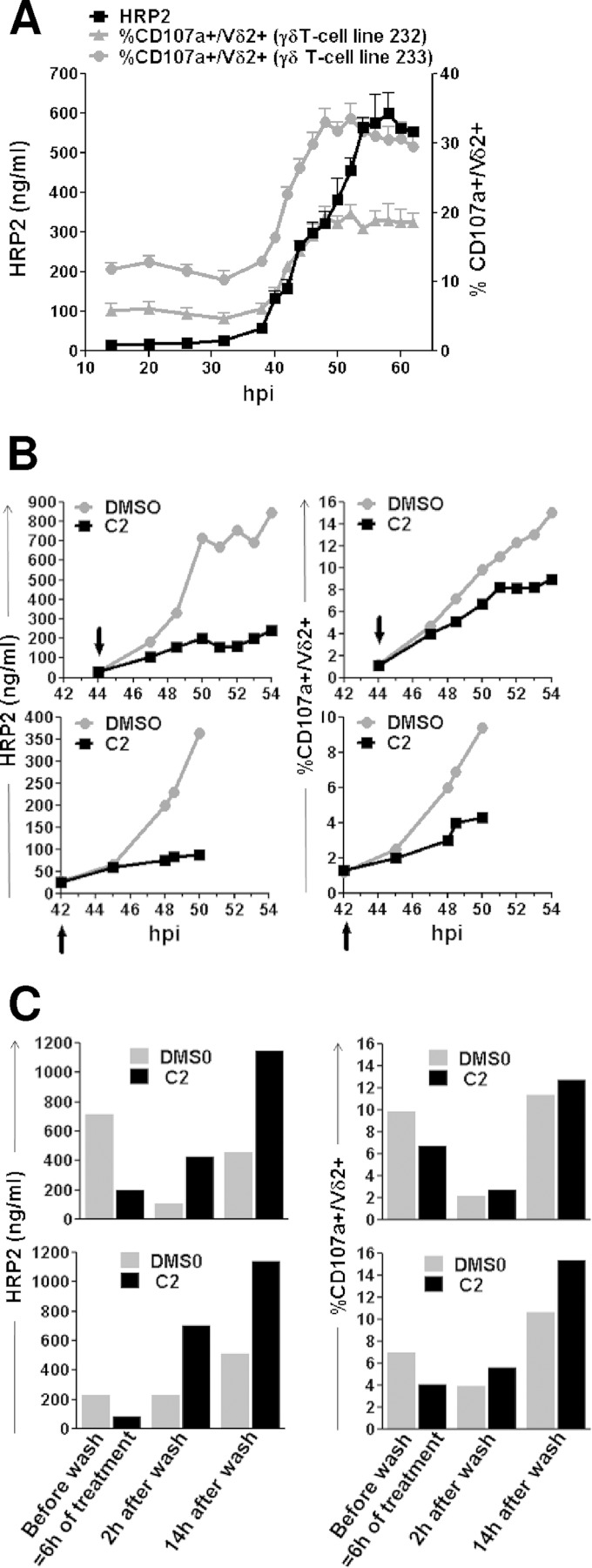
Phosphoantigens are released during iRBC rupture. (A) Synchronized parasites from 3 independent cultures (A, B, and C) at 1% parasitemia were cultured in CPM for 62 h, and iRBC supernatants were collected at the indicated time points (hours postinvasion [hpi]) across the parasite developmental cycle (time zero corresponds to parasitic invasion). The phosphoantigen bioactivity in the various iRBC-SNs was assessed on two independent γδT-cell lines (232 and 233) using CD107a surface expression. HRP2 concentration in the iRBC-SNs was determined by ELISA. Data show the means ± SD from CD107a expression induced by the three independent culture SNs (A, B, and C) and their means ± SD for HRP2 content at each time point. (B) Synchronized iRBC cultures (1% parasitemia) were treated with compound 2 (C2) or a control (DMSO) at the time indicated by the arrow, and supernatants were collected at different time points of treatment. Two independent C2 treatment experiments are shown on 44-hpi schizonts (top) and 42-hpi schizonts (bottom). HRP2 content was measured by ELISA (left), and phosphoantigen concentrations were assessed by CD107a test (right) on 3 γδT-cell lines. Results for representative γδT-cell line 384 are shown. (C) Parasite cultures used for panel B were washed after 6 h of treatment with compound (C2), fresh medium was added, and supernatants were collected at the indicated times postwash and tested for both their HRP2 content (left) and their ability to induce Vγ9Vδ2 T-cell degranulation (right). Shown are results from one γδT-cell line (the same as that shown in panel B) out of three.

The results described above were obtained with iRBC culture supernatants collected from synchronized cultures containing mostly mature stages (38 to 40 hpi) of the parasite. To investigate the kinetics of activator molecule release during blood-stage development, supernatants were collected at different time points after invasion and their bioactivity was tested on two γδT-cell lines using the CD107a assay (Fig. 4A). The timing of iRBC schizont rupture was evaluated by monitoring in parallel the release of HRP2 into the iRBC supernatants, which occurs at the time of parasite egress (38). Bioactivity in the supernatants raised abruptly around 40 hpi and reached a plateau by 50 hpi. Comparable activation kinetic profiles were observed for the two γδT-cell lines, although maximal CD107a reactivity was 20% for γδT-cell line 232 and more than 30% for γδT-cell line 233. The kinetics of HRP2 release were superimposable on those of bioactivity release and were consistent with schizont rupture monitored using Giemsa-stained smears, which showed reinvasion starting at approximatively 40 hpi and being essentially complete by 50 hpi. Altogether, this indicated that the Vγ9Vδ2 activators were released concomitantly with HRP2, at the very end of the intraerythrocytic cycle, most likely upon parasite egress.

To confirm this conclusion, we blocked parasite egress using compound 2 (C2), a specific inhibitor of the parasite cGMP-dependent protein kinase G that regulates parasite egress (35). After checking that the C2 used did not affect Vγ9Vδ2 T-cell activation by HMBPP, we tested the ability of supernatants from C2-treated cultures to activate Vγ9Vδ2 T cells (Fig. 4B). Late intraerythrocytic developmental stages were treated with C2 for up to 10 h, and the culture supernatants collected at different time points were tested for HRP2 and Vγ9Vδ2 T-cell activation capability. We performed experiments with parasites at approximately 44 hpi (Fig. 4B, top) or parasites at approximately 42 hpi to avoid early schizont rupture events (Fig. 4B, bottom). Under both conditions, blocking schizont rupture by C2 prevented the release of HRP2 into the culture supernatant, as expected, and strongly decreased their bioactivity (Fig. 4B), leading to a C2-induced plateau, contrasting with the sustained increase in the mock-treated culture. C2 treatment was reversible, as parasites resumed egress and simultaneously discharged HRP2 and phosphoantigens into the supernatants after C2 withdrawal (Fig. 4C). There was an almost quantitative recovery of the HRP2 and phosphoantigen bioactivity in the C2-treated culture supernatant after washing out C2 and further culturing the parasites in the absence of C2. Altogether, these data led to the conclusion that the bulk of the activator molecule content is released upon schizont rupture, although we cannot exclude leaking of some bioactivity due to increased permeability of iRBCs at the latest stages of schizogony (39).

### Quantification of phosphoantigen bioactivity.

**FIG 5:**
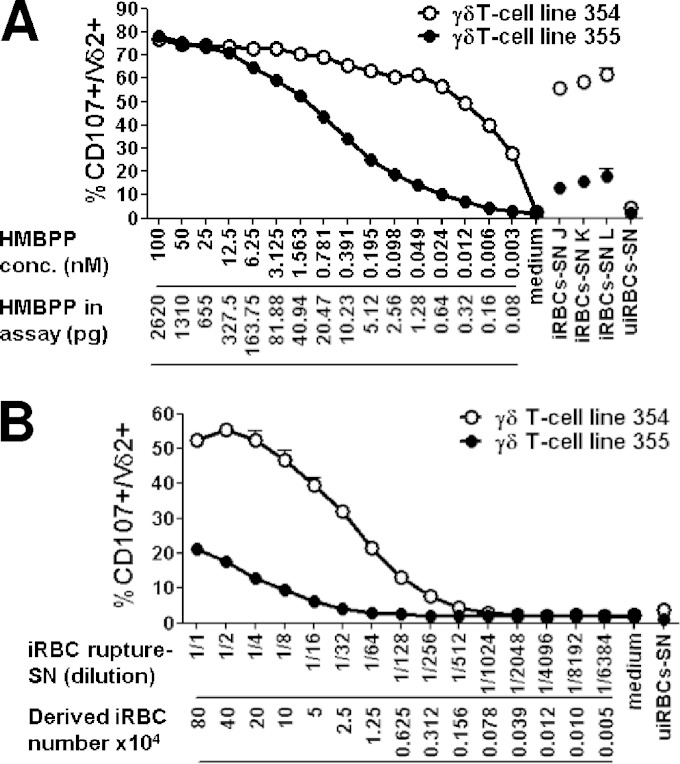
Measurement of Vγ9Vδ2 T-cell stimulation bioactivity in iRBC supernatants. Dose-response stimulation of two different γδT-cell lines with either HMBPP expressed as a concentration and corresponding quantities in the assays (A) or supernatant of fully ruptured iRBCs (see Materials and Methods) and the theoretical corresponding number of contributing iRBCs in the assay, extrapolated from the dilution factor (B). After stimulation, CD107a surface expression was assayed. Vγ9Vδ2 T-cell activation obtained with culture supernatants J, K, and L and uiRBC supernatant are shown. Data represent means ± SD from duplicates. Titration of the rupture supernatant with one γδT-cell line was calculated at half of the maximum of the percent CD107a^+^ Vδ2^+^, which was obtained after stimulation by both supernatant dilution determined in panel B and the HMBPP concentration determined in panel A. The HMBPP equivalent content of the supernatant corresponds to the HMBPP concentration determined in panel A multiplied by the supernatant dilution factor determined in panel B.

Due to insufficient sensitivity, we were not able to directly measure phosphoantigen concentrations in the supernatants by standard techniques (40). Therefore, we quantified bioactivity in iRBC supernatants using the CD107a assay with two different γδT-cell lines. We expressed bioactivity as HMBPP equivalents using a calibration curve of Vγ9Vδ2 T-cell activation obtained with HMBPP. Interestingly, the two γδT-cell lines prepared freshly for this experiment had different dose-response profiles from that for HMBPP (Fig. 5A). γδT-cell line 354 was exquisitely responsive, with a maximal degranulation plateauing at concentrations above 0.39 nM HMBPP and with a calculated 50% maximal response concentration (EC_50_) of 0.005 nM. γδT-cell line 355 was less responsive, with a calculated EC_50_ of 0.571 nM HMBPP and a response threshold of 0.012 nM. The bioactivity of supernatants J, K, and L, collected from late schizont cultures (previously used in experiments shown in Fig. 2), was fitted to these dose-response curves. J, K, and L bioactivities were estimated at 0.029, 0.044, and 0.072 nM HMBPP equivalents, respectively, using γδT-cell line 354, and at 0.042, 0.063, and 0.084 nM HMBPP equivalents, respectively, using γδT-cell line 355. This outlined a similar ability to detect phosphoantigens for both γδT-cell lines, despite differing sensitivity and threshold responses.

To evaluate the overall parasite bioactivity, we collected the rupture supernatant from a culture in which schizont rupture, parasite egress, and parasite invasion had proceeded to completion. Bioactivity of this rupture supernatant was titrated with the same two 354 and 355 γδT-cell lines and found to be 0.100 nM and 0.192 nM, respectively (Fig. 5B). This indicated that 80 × 10^4^ ruptured schizont iRBCs produce roughly 2.6 to 5.2 pg HMBPP equivalents, i.e., 0.003 to 0.006 fg per iRBC. Altogether, these results show that Vγ9Vδ2 T cells are extremely sensitive to trace amounts of phosphoantigens.

## DISCUSSION

In this paper, we clarify how Vγ9Vδ2 T cells are activated by blood-stage malaria parasites. We show that iRBC-Vγ9Vδ2 T-cell contact is dispensable, although we cannot formally exclude a possible interaction with merozoites. This is consistent with the absence of BTN3A on the iRBC surface, and that molecules with the characteristics of phosphoantigens, the Vγ9Vδ2 T-cell-activating moieties, are produced as soluble molecules from the DOXP pathway and released mostly at the time of P. falciparum erythrocytic egress. Vγ9Vδ2 T cells are shown to be exquisitely sensitive to phosphoantigen stimulation, although with a substantial variability among donors. The estimated parasite bioactivity and the low threshold of Vγ9Vδ2 T-cell activation are compatible with a distant activation of Vγ9Vδ2 T cells by phosphoantigens released in the blood during malarial infection.

As BTN3A is not expressed onto iRBCs, phosphoantigens cannot be presented to Vγ9Vδ2 T cells by this molecule by the iRBCs, unlike cancer cells (21–24). The potent activation noted by soluble molecules released in the culture supernatant by iRBCs indicates that presentation by the iRBC itself is fully dispensable. Ultracentrifugation ruled out the possibility of presentation by microvesicles (36), and efficacy of BTN3A blocking on Vγ9Vδ2 T-cell activation suggested an autopresentation of the captured parasite-derived activator molecules by the Vγ9Vδ2 T cells that express BTN3A (25, 41). Thus, our overall results showed that the activating molecules have all the characteristics of phosphoantigens.

The timing of expression of the DOXP pathway in the apicoplast (42, 43) is consistent with the observed stage-dependent bioactivity, as young intracellular stages (ring stages and young trophozoites), in which the apicoplast is poorly active, do not stimulate Vγ9Vδ2 T cells (44). The exact chemical composition of parasite stimulants still is uncertain; this is why the quantification of bioactivity was estimated against an HMBPP reference, which is the most active phosphoantigen reported to date. Accordingly, our quantification of soluble bioactivity, expressed as HMBPP equivalents, may underestimate the actual amount of activators if some, such as IPP (15), have a lower specific activity. Nevertheless, our estimates are in accordance with the range of bioactivity (0.1 to 10 nM) measured in supernatants of patient's neutrophils that phagocytosed HMBPP-producing bacteria (45), with these amounts being sufficient to stimulate Vγ9Vδ2 T cells.

Essentially similar temporal patterns of HRP2 and Vγ9Vδ2 T-cell stimulant release into the microenvironment were observed (Fig. 4A). The bulk of these stimulants seem to be released at the end of schizogony, when the parasite egresses from the erythrocyte. Inhibition of their release by C2 was somewhat less efficient when parasites were treated at the very late developmental stages (Fig. 4B, upper). This suggests some leaking of phosphoantigens before egress, likely due to the increased permeability of iRBCs at the later stages of schizogony, as suggested by recent observations showing permeability to immunoglobulins (39). In this case, leakiness would preferentially affect low-molecular-mass metabolites, such as phosphoantigens, while proteins of the size of HRP2 (approximately 35 kDa) would remain intracellular. Several studies, including ours, reported stimulation of Vγ9Vδ2 T cells by intact mature iRBCs cocultivated with Vγ9Vδ2 T cells (1, 5, 9, 14, 30). We calculated that the amount of stimulants released from as few as 2% of the iRBCs for the first 20 h of parasite culture already could be above the threshold of Vγ9Vδ2 T-cell activation. This suggests that at least part of stimulation by so-called intact iRBCs occurs through phosphoantigens released from the iRBCs during cocultivation, either upon artifactual, spontaneous lysis of fragile iRBCs or rupture of a few older schizonts in the parasite culture. Nevertheless, the conclusion that the bulk of phosphoantigens are released upon schizont rupture is substantiated by the fact that supernatants collected after complete rupture (Fig. 5B) yielded a 10-fold larger amount of bioactivity than supernatants collected from late developmental stages. As the supernatants were filtered, the potential contribution of direct activation of Vγ9Vδ2 T cells by egressed merozoites is excluded.

There was some variability of γδT-cell line sensitivity to parasite supernatants, which were used undiluted in most experiments. This differed from the homogeneously maximal activation conveyed by 100 nM HMBPP, used as a positive control. Variability of the response to HMBPP itself was readily unmasked when using concentrations several logs lower than 100 nM (Fig. 5A), i.e., in the range of bioactive phosphoantigens produced by iRBCs. γδT-cell line variability, highlighted at low stimulating concentrations, might be explained by donor-dependent variability of circulating Vγ9Vδ2 T cells among PBMC (TCR density, differentiation status, immunologic history, and current infections) or could reflect variability generated during the *in vitro* expansion of Vγ9Vδ2 T cells for 20 to 22 days. Donor-dependent variability of IFN-γ production by Vγ9Vδ2 T cells in response to iRBCs has been reported by d'Ombrain et al. (30).

The experimental conditions used here to investigate the dose response of Vγ9Vδ2 T cells (10^5^ cells and up to 80 × 10^4^ iRBCs) allow some extrapolation to clinical situations. In malarial patients, parasite counts in the range of 80 × 10^4^ iRBCs/ml (0.02% parasitemia) are frequently observed. Such numbers should release enough phosphoantigens to distantly stimulate patrolling Vγ9Vδ2 T cells *in vivo*. As mature P. falciparum intraerythrocytic stages are sequestered in the microvasculature (46), schizont burst occurs in anatomically specific niches. We speculate that the elevated sensitivity of Vγ9Vδ2 T cells allows *in vivo* activation despite dilution of phosphoantigens in the extracellular milieu and/or in the bloodstream. Triggering of Vγ9Vδ2 activation could occur in the red pulp of the spleen, where Vγ9Vδ2 T cells accumulate and young intraeythrocytic stages are retained in the slow open circulation (47). It also could occur in microvessels, where mature iRBC sequestration reduces the blood flow and provokes infiltrates and inflammation (46).

Exploring these hypotheses is complicated by the inappropriate sensitivity of phosphoantigen detection in patient's plasma using mass spectrometry, as the reported limit of quantification for DMAPP and IPP is in the range of 30 nM (40), i.e., 2 to 3 orders of magnitude less than that of the Vγ9Vδ2 T cells in bioassay used here. Our preliminary investigations did not find phosphoantigen bioactivity in malaria patients' plasma. This may reflect the short half-life of phosphoantigens in the peripheral circulation (48) and does not exclude high concentrations in some tissues.

The results presented here provide a novel framework to understand the activation of Vγ9Vδ2 T cells during malaria infection and, more generally, infection by microorganisms lacking butyrophilin and releasing or secreting activator molecules such as phosphoantigens into the microenvironment.

## Supplementary Material

Supplemental material
