# Supplementary material for: Phosphoantigen Burst upon Plasmodium falciparum Schizont Rupture Can Distantly Activate Vγ9Vδ2 T Cells
Source: Infect Immun. 2015 Sep 10;83(10):3816–24. doi: 10.1128/IAI.00446-15 (PMC4567633; doi:10.1128/IAI.00446-15)
Supplement: Supplemental material [file supp_83_10_3816__index.html]

Phosphoantigen Burst upon Plasmodium falciparum Schizont Rupture Can Distantly Activate Vγ9Vδ2 T Cells — Supplemental material 

# Phosphoantigen Burst upon Plasmodium falciparum Schizont Rupture Can Distantly Activate Vγ9Vδ2 T Cells

## Supplemental material

- Supplemental file 1 -

  Fig. S1. Vγ9Vδ2 T cell activation by iRBCs does not require contact. Fig. S2. Rescue of fosmidomycin-treated parasites with FPP.

  PDF, 241K
