# Supplementary material for: Phosphoantigen Burst upon Plasmodium falciparum Schizont Rupture Can Distantly Activate Vγ9Vδ2 T Cells
Source: Infect Immun. 2015 Sep 10;83(10):3816–24. doi: 10.1128/IAI.00446-15 (PMC4567633; doi:10.1128/IAI.00446-15)
Supplement: Supplemental material [file IAI.00446-15_zii999091396so1.pdf]

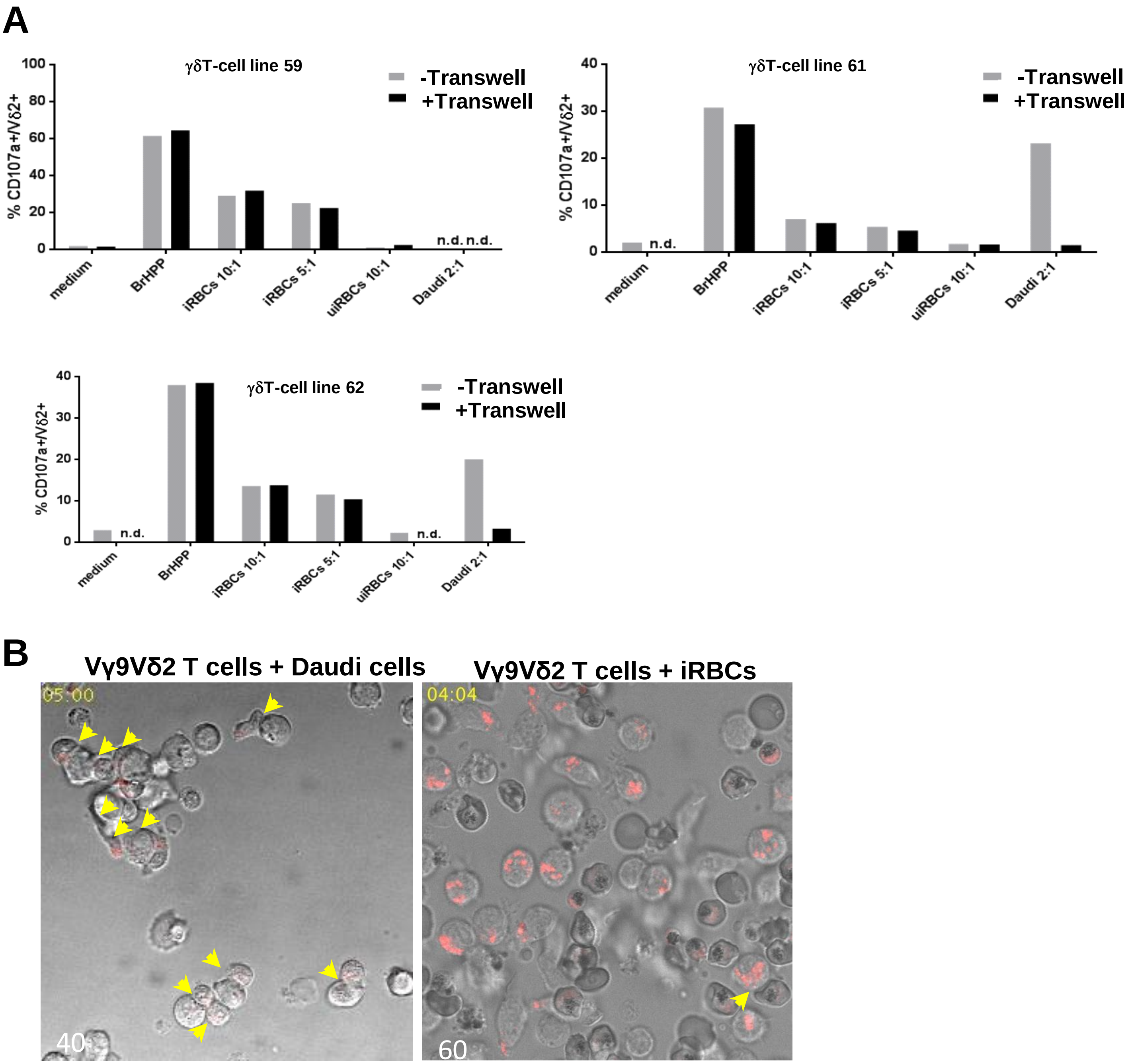

Figure S1. Vγ9Vδ2 T cell activation by iRBCs does not require contact. (A) Vγ9Vδ2 T cell degranulation was assessed by the CD107a assay after 4h-incubation of γδT-cell lines with stimulants present in the same well (grey bars) or separated by a Transwell device (black bars). BrHPP was used as a soluble, freely diffusing positive control. Daudi cells were used as a non diffusing stimulant control. Different iRBC/ γδT-cell ratios were used as indicated. uiRBC refers to uninfected red blood cells. Nd: not done. Experimental results from 3 different γδT-cell lines (59, 61 and 62) are shown. **(B)** Highly purified Vγ9Vδ2 T cells (>90% of purity) which had been screened for capacity to degranulate in the presence of iRBCs with an average reactivity >50%, were labeled with Lysotracker red and incubated with purified iRBCs (38 hpi) at different effector:target ratios or with Daudi cells (E:T ratio = 1:2) as control. During the 2 hours of observation, almost all Vγ9Vδ2 T cells formed long-lived conjugates with Daudi cells, while iRBC-Vγ9Vδ2 conjugates could scarcely be observed. Freeze screen time-lapse microscopy pictures were captured at 5:00 min (Daudi cells) or 4:04 min (iRBCs) using a Leica DMI6000 TCS SP5, confocal microscope inverted stand and lasers emitting at 488 and 543 nm. Images were processed using ImageJ software.

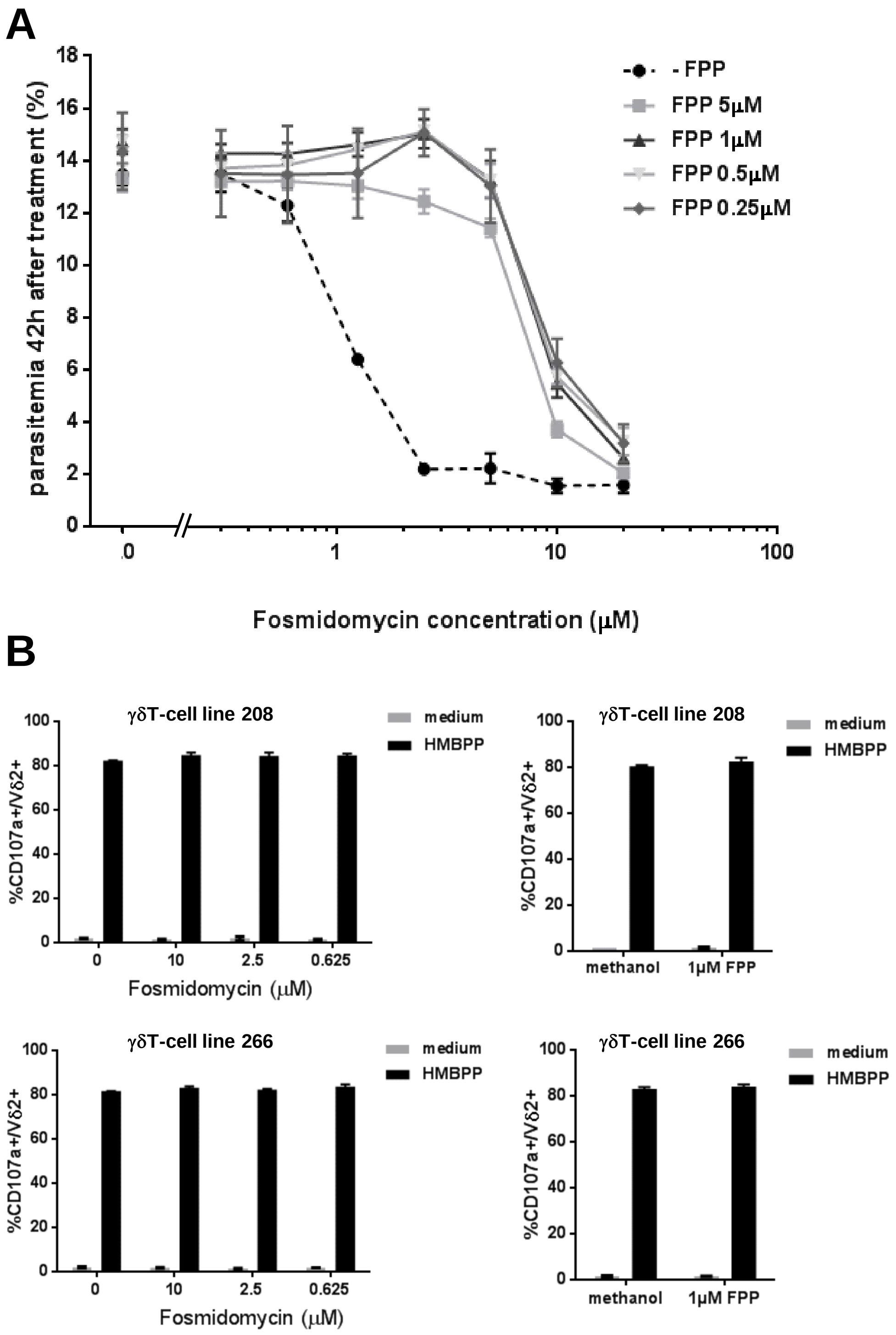

Figure S2. Rescue of fosmidomycin treated parasites with FPP. (A) Parasite cultures were treated with increasing doses of fosmidomycin alone (doted lines) or with FPP added at various concentrations (full lines curves). Controls in CPM alone, without fosmidomycin and with FPP are shown on the left. Parasitemia was measured 42 hours post-treatment by flow cytometry using hydroethidine staining. **(B)** To check that fosmidomycin and FPP have no effect on Vγ9Vδ2 T cell activation, Vγ9Vδ2 T-cell degranulation was tested in cell culture medium with or without 100 nM HMBPP, in the presence of fosmidomycin (left panels) or 1 µM FPP (right panels) or its equivalent solvent (methanol). Means+/-SD are shown from duplicate supernatants and on two different γδT-cell lines.
